# How to choose optimal adjuvant therapies for high-risk hormone receptor-positive, HER2-negative breast cancer after chemotherapy?

**DOI:** 10.2340/1651-226X.2025.43645

**Published:** 2025-06-25

**Authors:** Peeter Karihtala

**Affiliations:** a Department of Oncology, Helsinki University Hospital Comprehensive Cancer Center and University of Helsinki, Finland; b Department of Oncology and Radiotherapy, Oulu University Hospital, Finland

**Keywords:** Adjuvant therapy, bisphosphonates, CDK4/6 inhibitors, disparities, endocrine treatment, PARP inhibitor

## Abstract

**Background and purpose:**

The prognosis for hormone receptor (HR)-positive, human epidermal growth factor receptor 2 (HER2)-negative breast cancer has significantly improved over the past few decades. However, a substantial number of patients still face an elevated risk of recurrence. Due to the high prevalence and cumulative mortality of HR+/HER2- breast cancer, it poses a global health challenge.

**Material and methods:**

This is a narrative review on the post-chemotherapy treatment options in patients with HR+/HER2- breast cancer.

**Results:**

Endocrine therapy remains the cornerstone of adjuvant treatment, with extended durations of tamoxifen and aromatase inhibitors demonstrating survival benefits. Several novel post-chemotherapy adjuvant treatments have recently been introduced for high-risk patients, and now most patients with HR+/HER2- breast cancer are eligible for non-endocrine adjuvant therapies. Bisphosphonates help to reduce bone recurrence and enhance overall survival in postmenopausal women, though the evidence remains somewhat inconsistent. CDK4/6 inhibitors abemaciclib and ribociclib have also emerged as adjuvant therapies, while the poly ADP ribose polymerase (PARP) inhibitor olaparib provides clinically meaningful benefits for patients with germline BRCA1/2 mutations.

**Interpretation:**

Optimal patient selection for these often toxic treatments remains partially unclear and is the focus of intensive research. In the near future, monitoring ctDNA may enable treatment de-escalation for selected high-risk patients. The rise of perioperative immunological therapies, new CDK4-specific inhibitors, and targeted endocrine treatments can lead to a notably favorable prognosis for many previously high-risk HR+/HER2- breast cancers. Future research should prioritize predictive biomarkers and personalized approaches to optimize treatment efficacy, ensure more equal access to treatments, and minimize overtreatment.

## Introduction

Hormone receptor (HR)-positive, human epidermal growth factor receptor 2 (HER2)-negative breast cancers, commonly referred to as luminal-like breast cancers, account for 70–80% of all breast cancer cases and are a major contributor to breast cancer-related deaths, presenting a global health challenge [1–3]. Overall, their prognosis is favorable and has improved in recent decades due to earlier diagnoses and enhanced adjuvant treatments, although substantial geographical variations in prognosis still exist [4–7]. However, despite modern treatments, distant recurrences are not uncommon, and the annual risk of death from HR+/HER2- breast cancer does not plateau even after decades of follow-up [5, 8]. Therefore, a clear, unmet need remains for innovative strategies to reduce the recurrence rates of these cancers.

The precise postoperative risk stratification is essential for planning risk-based adjuvant treatments and organizing adequate surveillance [9]. Lymph node involvement and tumor size are the most established factors predicting subsequent recurrence also in HR+/HER2- breast cancer [10, 11]. The prognostic role of Ki-67 has long been debated, primarily due to technical issues regarding analytical validity. Still, clear evidence indicates that a higher Ki-67 labeling index is associated with worse outcomes, independent of other prognostic factors [12–14]. There is still no unequivocal consensus on the cut-off for low or high Ki-67; but for example the European Society for Medical Oncology (ESMO) Early Breast Cancer Guidelines use a Ki-67 expression of ≥ 20% as a cut-off for high-risk disease and also for classifying luminal A-like and luminal B-like breast cancers [15]. Another surrogate marker for distinguishing luminal A-like from luminal B-like breast cancers, progesterone receptor expression, is also an independent prognostic factor for HR+/HER2- breast cancer, particularly in premenopausal women [10, 16]. Lastly, both histological grade and histological subtypes are established prognostic factors for HR+/HER2- breast cancers, with grade 3 tumors especially linked to higher recurrence rates and shorter survival times, while tubular, mucinous, and cribriform histological subtypes indicate more favorable outcomes [10, 17, 18]. Invasive lobular carcinomas (ILC) usually show the HR+/HER2- subtype and a lower grade than the most common invasive subtype, invasive carcinoma of no special subtype (NST) [18]. While in the most published post-adjuvant chemotherapy trials invasive carcinomas NST have consisted by far the majority, there is clear evidence that ILC responds on average poorly to chemotherapy, but well to endocrine therapy. In contrast to all the other risk factors mentioned above, which often play a role in the risk stratification of breast cancer adjuvant trials, histological type has rarely been a stratification factor or inclusion/exclusion criterion. Although specific histological subtypes with favorable outcomes have been presented as a basis for de-escalation in international guidelines, more randomized data on how specific drugs affect tumors with particular histological subtypes are needed [15, 20].

Most high-risk HR+/HER2- breast cancer patients are recommended to undergo neoadjuvant systemic therapy before surgical treatment [15, 20]. While pathological complete response (pCR) serves as a less established surrogate for long-term survival in HR+/HER2- breast cancer compared to other subtypes, it still helps to identify patients at a higher risk of relapse and can be used [21, 22, 139]. For instance, the response to neoadjuvant chemotherapy can be used to identify patients who may benefit from adjuvant treatment with olaparib [22]. Several genomic signatures have been able to predict the risk of distant metastases for up to 10 years in HR+/HER2- breast cancer, particularly OncotypeDX, which carries the highest level of evidence [23–29]. Although these genomic signatures are not suitable for evaluating the effectiveness of new adjuvant therapies, they have been useful in stratifying risk in contemporary adjuvant clinical trials.

Due to current unmet needs, several novel approaches have been introduced over the last decade to reduce the risk of recurrence in HR+/HER2- breast cancer. Most studies evaluating these new medications have already reported substantial follow-up times and also clinically meaningful survival benefits. There are currently several treatment options available to clinicians, but all come with frequent toxicity. Additionally, comparative clinical studies on different approaches are still lacking. Therefore, the question is not only what to add, but also in which patients, even with the high-risk features in their tumors, there are opportunities to de-escalate treatments. This review examines optimal adjuvant therapies for high-risk HR+/HER2- breast cancer following chemotherapy, highlighting the necessity for personalized and risk-stratified strategies. Contemporary evidence from neoadjuvant and adjuvant chemotherapies has been discussed in other reviews [30, 31].

## Methods

This article is a narrative review aimed at summarizing and critically interpreting current evidence on adjuvant treatment strategies for patients with high-risk HR+/HER2- early breast cancer after chemotherapy. The primary objective was to provide clinically meaningful guidance for optimizing treatment selection in this patient population, integrating both landmark and emerging studies.

Relevant literature was identified through a comprehensive search of the PubMed/MEDLINE database, ClinicalTrials.gov, and major oncology conference abstracts (American Society of Clinical Oncology [ASCO], ESMO, San Antonio Breast Cancer Symposium [SABCS]) up to March 2025. Search terms included combinations of ‘breast cancer’, ‘HR-positive’, ‘hormone-receptor-positive’, ‘ER-positive’, ‘estrogen receptor-positive’, ‘HER2-negative’, ‘adjuvant therapy’, ‘endocrine therapy’, ‘CDK4/6 inhibitor’, ‘bisphosphonate’, ‘bone-modifying agent’, ‘PARP inhibitor’, ‘barriers’, and ‘risk stratification’. Both clinical trial reports and high-impact review articles were included. Reference lists of retrieved articles were manually screened to identify additional relevant publications.

Priority was given to randomized controlled trials, meta-analyses, and phase III studies with mature follow-up, although pertinent phase II studies and subgroup analyses were also considered. Studies were included regardless of geographic origin, provided they were published in English. Articles focusing solely on metastatic disease or on neoadjuvant chemotherapy without addressing post-chemotherapy adjuvant strategies were excluded.

This review does not aim to provide a systematic evaluation or meta-analysis of all available data but instead focuses on integrating pivotal clinical findings with an emphasis on clinical value. Emerging therapeutic concepts and ongoing trials were included selectively to highlight promising directions and areas of uncertainty. The included evidence was critically appraised for clinical relevance, strength of outcomes (e.g. survival vs. surrogate endpoints), and applicability to routine care.

## Thematic review

### Intensifying endocrine treatment

**Figure 1:**
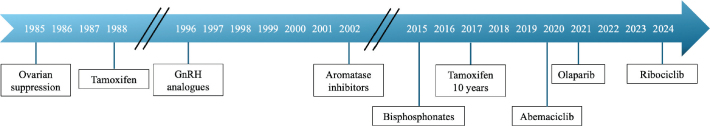
A timeline highlighting the original publication year of practice-changing therapeutic options in early-stage HR+/HER2- breast cancer.

**Figure 2:**
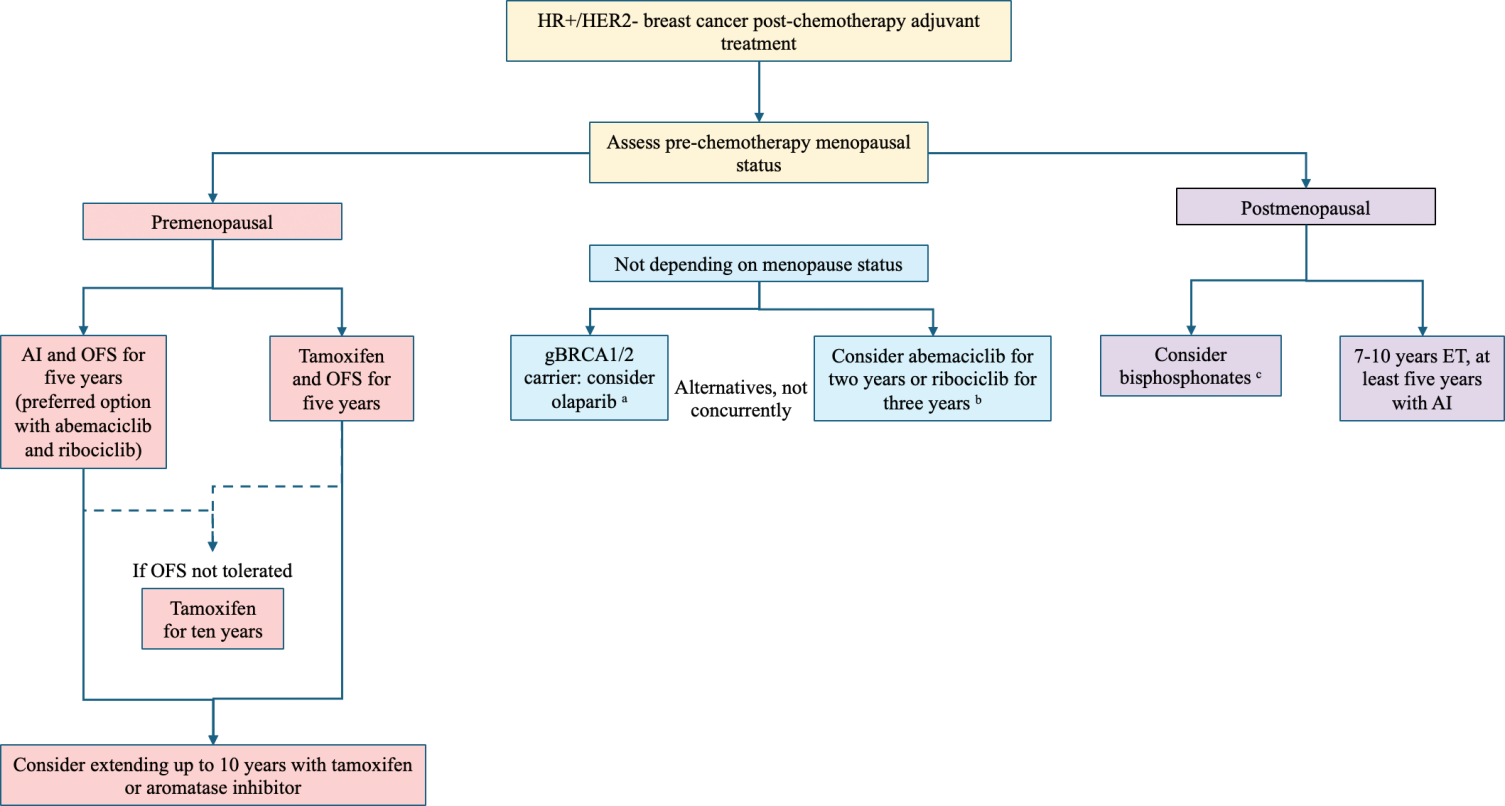
A flow diagram showing possible treatment options for high-risk, early-stage HR+/HER2- breast cancer patients after chemotherapy. ^a^The inclusion criteria for the OlympiA study have been published by Tutt et al. [22]. ^b^The approval for CDK4/6 inhibitors varies between regulatory authorities. ^c^Regarding guideline recommendations for specific bisphosphonates and dosing, please see the text for more details. AI: aromatase inhibitor; ET: endocrine therapy; OFS: ovarian function suppression; TAM: tamoxifen; HER2: human epidermal growth factor receptor 2.

After 120 years of anti-hormonal treatment for breast cancer and more than 50 years since the introduction of tamoxifen, the first targeted therapy in breast cancer, endocrine treatment remains the backbone of adjuvant therapy for ER+/HER2- breast cancer (Figures 1 and 2) [32, 33]. While the 5-year duration of endocrine treatment is standard for most patients, those with high-risk tumor features benefit significantly from extended periods of adjuvant endocrine treatment. Tamoxifen, a selective estrogen receptor modulator, notably reduces invasive recurrences and improves overall survival, demonstrating a significant carry-over effect even when administered for 2 or 5 years postoperatively [34–38]. When comparing 10 years of adjuvant tamoxifen to the standard 5 years, recurrences decrease by 16%, and overall survival increases by 13%, with absolute benefits of 3.7 and 3.8% points, respectively [39]. Extrapolating these findings to the early studies with no tamoxifen treatment at all, 10 years of tamoxifen can cut breast cancer-related mortality in half compared to no adjuvant endocrine therapy [35, 36]. The absolute benefit is most pronounced in women at the highest risk, prompting many guidelines to recommend 10 years of endocrine treatment for women with high-risk features in their cancer, such as node positivity or a primary tumor size greater than two centimeters [10, 15]. This escalation must be balanced against the tolerability of the drug during the initial 5 years of tamoxifen, as 36 patients need to receive and an additional 5 years of tamoxifen to prevent one breast cancer-related death [39].

Also postmenopausal women with HR+/HER2- breast cancer can face an elevated risk of cancer-related death for decades. Given that aromatase inhibitors have shown survival benefits over tamoxifen in the adjuvant setting, extending treatment durations with aromatase inhibitors has been explored [40]. In the phase III MA.17 trial (*n* = 5,170), patients were randomized to receive either 5 years of letrozole or 5 years of placebo after completing 5 years of tamoxifen treatment. Letrozole significantly decreased distant recurrences (hazard ratio [HR] 0.60, 95% confidence interval [CI] 0.43–0.84), and node-positive patients also demonstrated a borderline significant overall survival benefit (HR 0.61, 95% CI 0.38–0.98) [41]. Similarly, in the NASBP B-33 trial, exemestane improved relapse-free survival (but not disease-free survival [DFS]) compared to placebo after 5 years of tamoxifen, although the study was closed prematurely following the publication of the MA.17 results [42]. Several studies suggest a modest benefit in DFS by extending aromatase inhibitor treatment duration to 7–10 years. However, about half of this benefit is attributed to a reduction in local recurrences, and none of the published studies have demonstrated improved overall survival with extending aromatase inhibitor therapy beyond 5 years [43–47]. Taken together, the strongest evidence for improving overall survival with extended adjuvant endocrine therapy comes from tamoxifen, with mixed evidence from tamoxifen-aromatase inhibitor sequencing, while there is no evidence supporting overall survival benefits from 7 to 10 years of aromatase inhibitors compared to 5 years. Unfortunately, from a clinical standpoint, no phase III studies have evaluated the sequence of adjuvant treatment starting with aromatase inhibitors followed by tamoxifen, although there are ongoing efforts to determine the optimal sequencing of tamoxifen and aromatase inhibitors in the extended adjuvant setting (NCT06223698).

Genomic assays and risk scores can help in refining patient selection for over 5 years of endocrine therapy. Clinical Treatment Score post-5 years (CTS5) integrates four clinicopathological variables and has been validated to predict late luminal breast cancer recurrences [48–50]. Gene expression assays can also flag patients at the highest risk for late relapse. The low-risk patients with EndoPredict (EPclin) assay have only a 3% risk of 10-year distant recurrence, while EPclin high-risk patients carry a much higher risk [51]. Although EndoPredict and another genomic assay, PAM50, have not been demonstrated yet to show which patients would benefit from extended endocrine therapy, in practice, high risk scores from these assays might support extending therapy, whereas a low score might argue against routine extension. No trial has demonstrated that the OncoType DX score would specifically predict the benefit from extended endocrine therapy. Instead, Breast Cancer Index (BCI) and MammaPrint assays have been shown being able to identify patients benefiting from longer durations of endocrine treatment [52, 53]. In a comparative analysis, PAM50, BCI and EndoPredict were significantly more prognostic for overall and late distant recurrence than CTS and OncoType DX [51]. However, this applied mainly to the node-negative patients, while information is scarce from node-positive patients.

For premenopausal patients, the most effective endocrine treatment combines ovarian suppression with either tamoxifen or exemestane. Mature data from large phase III trials, SOFT and TEXT, demonstrate that the effect of ovarian suppression is comparable to the efficacy of adjuvant chemotherapy [54, 55]. In both trials, the primary aim was to evaluate whether adding ovarian suppression to adjuvant endocrine treatment improves prognosis, particularly in women at high risk of recurrence. SOFT compared (1) tamoxifen alone with (2) tamoxifen plus ovarian function suppression (OFS) and (3) exemestane plus OFS, while TEXT focused on comparing (1) exemestane plus OFS with (2) tamoxifen plus OFS to identify the optimal endocrine therapy with ovarian suppression [55]. Patients in TEXT received immediate OFS, whereas those enrolled in SOFT may have undergone chemotherapy prior to ovarian suppression. The 12-year results from SOFT indicated an overall survival benefit of 5.6% points with OFS among women who received adjuvant chemotherapy [55]. The combined analysis of SOFT and TEXT, which included data from over 5,600 women, showed an overall survival benefit of 4.5% points after a 13-year follow-up for exemestane plus ovarian suppression compared to tamoxifen plus ovarian suppression in women with high recurrence-risk features (age under 35, tumor size over 2 cm, or grade 3) [54].

**Figure 3:**
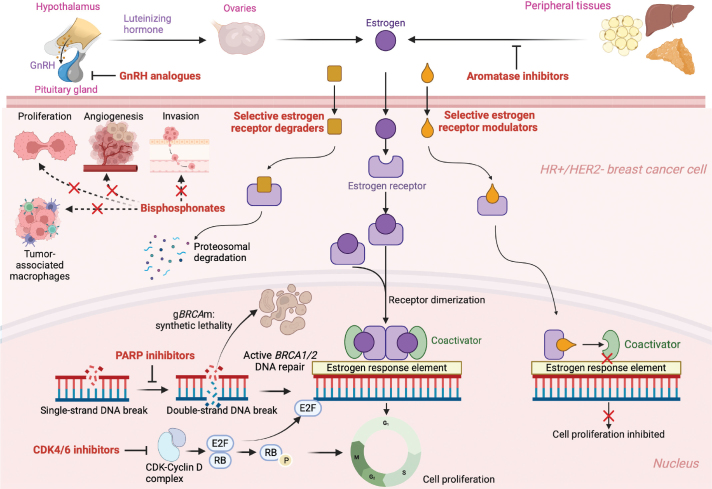
A schematic illustration of the mechanism of action of adjuvant treatment options in HR+/HER2- breast cancer after chemotherapy. Selective estrogen receptor degraders are shown as an example of potential future therapeutic options, but they are not indicated in the treatment of early breast cancer. CDK: cyclin-dependent kinase; GnRH: gonadotropin-releasing hormone. Figure created with bioRender.

The first-generation selective estrogen receptor degrader (SERD) fulvestrant did not provide a benefit over standard adjuvant endocrine treatment [56]. The second-generation SERDs allow for oral administration and demonstrate improved pharmacokinetics as well as greater potency against mutated estrogen receptor-alpha (Figure 3) [57, 58]. The favorable toxicity profile of novel SERDs is also encouraging, as poor adherence is often a concern even with 5-year endocrine treatments and is associated with worse prognosis [59–61]. Elacestrant has already been approved for treating metastatic breast cancer, and several phase III trials are currently evaluating whether the second-generation SERDs will outperform standard endocrine treatment, either upfront or as an extended option after various durations of standard endocrine treatment (Table 1). It remains unclear whether novel SERDs will benefit only a specific subgroup of postmenopausal women, such as those with ESR1 mutations in their cancer cells, as observed in the metastatic setting [62]. For premenopausal patients without ovarian suppression, tamoxifen seems to remain the only adjuvant therapy option for the foreseeable future.

**Table 1:** Ongoing phase III trials, which evaluate the efficacy and safety of selective estrogen receptor degraders as breast cancer adjuvant therapy.

Trial acronym, NCT number and sponsor	Study arms	Intervention duration	Previous ET duration	Patient population	Primary endpoint	CDK4/6 inhibitor and PARP inhibitor treatment	Recruitment status, estimated study completion
lidERA NCT04961996, Hoffmann-La Roche	Giredestrant versus TAM/AI	Giredestrant 5 years (other ET as per investigator discretion)	≤ 12 weeks	Medium to high-risk, stage I–III	IDFS (excluding second non-primary BC)	Prohibited	Recruitment completed, estimated completion in 2033
CAMBRIA-1 NCT05774951, AstraZeneca	Camizestrant versus TAM/AI	5 years	21–63 months	Medium to high-risk, stage I–III	IBCFS	Permitted	Recruiting, estimated completion in 2036
CAMBRIA-2 NCT05952557, AstraZeneca	Camizestrant versus TAM/AI	7 years	≤ 12 weeks	Medium to high-risk, stage I–III	IBCFS	CDK4/6 inhibitor permitted, PARP inhibitor unknown	Recruiting, estimated completion in 2037
EMBER-4 NCT05514054, Eli Lilly and Company	Imlunestrant versus TAM/AI	5 years	2–5 years	Medium to high-risk, stage I–III	IDFS excluding second non-breast primary invasive cancers	Permitted	Recruiting, estimated completion in 2032
ELEGANT NCT06492616, Stemline Therapeutics	Elacestrant versus TAM/AI	5 years	2–5 years	Node-positive, high-risk	IBCFS	Permitted	Recruiting, estimated completion in 2032

*Source*: Data retrieved from www.clinicaltrials.org.

AI: aromatase inhibitor; BC: breast cancer; ET: endocrine therapy; IBCFS: invasive breast cancer-free survival; IDFS: invasive disease-free survival; NCT: National Clinical Trial; TAM: tamoxifen.

### Bone-modifying medications

Bisphosphonates are indicated for the treatment of osteoporosis, but aside from their bone-modifying properties, they also promote long-term activation of T-cells and tumor-infiltrating lymphocytes, while mitigating proliferation, invasion, and -angiogenesis [63–65]. Bisphosphonates display several anti-angiogenic properties and have potential to eliminate dormant cancer cells in the bone microenvironment [66, 67]. The use of bisphosphonates as adjuvant therapy for breast cancer was extensively researched during the early 2000s. With diverse populations and various bisphosphonates studied in each trial, the results were frequently contradictory. Most clinical guidelines incorporated bisphosphonates in adjuvant treatment following the Early Breast Cancer Trialists’ Collaborative Group (EBCTCG) meta-analysis in 2015 and subsequent meta-analyses [68–70]. In the EBCTCG meta-analysis, a slight reduction in bone recurrence was observed in the entire population (relative risk 0.83, 95% CI 0.73–0.94). Among postmenopausal women, this difference was notably more pronounced, yielding significant improvements in overall survival [10-year event rate 21.1% vs. 23.0%) and breast cancer-specific survival (10-year event rate 14.7% vs. 18.0%) for women receiving bisphosphonates (Table 2 [68]). The results remained consistent, regardless of ER status.

**Table 2:** Practical implications of the post-chemotherapy non-endocrine adjuvant treatments in HR+/HER2- breast cancer.

Medication	Target population	Starting dose and duration	Time of initiation	Overall survival benefit with statistical significance	ESMO MCBS	Number needed to treat to avoid one invasive recurrence at 4 years
Abemaciclib [85]	N2–N3 or T3N1 or N1 with grade 3 (in certain areas also N1 and Ki-67 ≥ 20%)	150 mg twice daily, along with ET for 2 years	After radiotherapy, with up to 12 weeks previous ET	No	A	20^a^
Ribociclib [86, 138]	Anatomical stage IIA, IIB or stage III	400 mg once daily, along with ET for 3 years	After radiotherapy, up to 12 months previous ET	No	A	20
Bisphosphonates^b^	Postmenopausal	Optimal duration not known^b^	Various approaches, no contraindications with surgery or other adjuvant treatments	Contradictory evidence from the original trials. In a meta-analysis (60), RR 0.82 (95% CI 0.73–0.93) in postmenopausal women	Not available	42 (in postmenopausal women, at 5 years in meta-analysis (68])
Olaparib [22, 139]	Germline *BRCA1/2* carriers After adjuvant chemotherapy: N2–N3 After neoadjuvant chemotherapy: no pCR and CPS+EG score ≥ 3	300 mg twice daily for 1 year	After radiotherapy	Yes, HR 0.68 (95% CI 0.47–0.97)	A	14

a At 5 years, the number needed to treat with abemaciclib to avoid one invasive recurrence is 13 ITT.

b Regarding guideline recommendations for specific bisphosphonates and dosing, please see the text for more detailed instructions. Bisphosphonates are officially approved for treating bone metastases, osteoporosis, and not for prevention of relapse.

CI: confidence interval; CPS + EG: clinical pathological staging-estrogen receptor grading; ET: endocrine therapy; HR: hazard ratio; ESMO MCBS: European Society for Medical Oncology Magnitude of Clinical Benefit Scale; pCR: pathological complete response; RR: risk ratio; HER2: human epidermal growth factor receptor 2.

There are several reasons why clinicians and clinical guidelines have interpreted the results from bisphosphonate adjuvant trials differently. These include the contradictory results between the studies, the exclusion of menopause status as a preplanned subgroup in the earliest trials, the combination of studies using different bisphosphonates, doses, and treatment durations in the meta-analyses, and a 3.3-fold increase in the risk of osteonecrosis of the jaw [69]. The current American Society of Clinical Oncology – Ontario Health (ASCO-OH) guidelines recommend discussing adjuvant bisphosphonate therapy with all postmenopausal patients who are candidates for adjuvant systemic therapy [71]. After the SUCCESS-A trial showed no benefit in extending zoledronic acid treatment beyond 2 years, ASCO-OH now also recommends a regimen of 4 mg once every 3 months for 2 years, along with peroral option with clodronate [71, 72]. Instead, NCCN guidelines recommend adjuvant bisphosphonates for 3–5 years to all high-risk early-stage breast cancer patients, including those who are node-negative [20]. The guidelines from ESMO are even more permissive, recommending bisphosphonates for women without ovarian function, ‘especially if at high risk of relapse’ [15]. According to these guidelines, it should not matter whether menopause is natural or therapy-induced, as indicated by the GnRH-analogue-treated patient population in some original studies. From a practical standpoint, the suboptimal adherence to GnRH-analogues is a well-recognized issue, particularly among the youngest patients [73, 74]. Since bisphosphonates can remain in the skeleton for decades [63, 75] and may lead to adverse outcomes in premenopausal patients [76], the safety of bisphosphonates in menopause-induced patient populations has been questioned at times. Furthermore, the EBCTCG meta-analysis showed that the benefit of adjuvant bisphosphonates was minimal in patients under 55 years [68]. Finally, a recent meta-analysis of bisphosphonate studies with contemporary adjuvant treatments suggested only a small DFS benefit (HR 0.89, 95% CI 0.81–0.97) and no overall survival benefit for bisphosphonates, although it was lacking individual patient data [77]. As it is unlikely that more large phase III studies will be conducted to assess the effects of bisphosphonates as breast cancer adjuvant therapy, clinicians must rely on the current, somewhat unusual situation in oncology, where the strongest clinical evidence derives from a meta-analysis. The NHS PREDICT calculator can also aid in evaluating the benefits of bisphosphonates, although it partly relies on older datasets [78].

The receptor activator of nuclear factor kappa-B (RANK) and its ligand (RANKL) axis play major roles in breast carcinogenesis, the remodeling of the cancer microenvironment, and the suppression of the immune system [79]. The RANKL inhibitor denosumab was studied in the phase III ABCSG-18 trial in the adjuvant setting for HR-positive patients, with a dosage of 60 mg every 6 months [80]. Compared to placebo, denosumab demonstrated a trend toward improved bone metastasis-free survival (HR 0.81, 95% CI 0.65–1.00) and overall survival (HR 0.80, 95% CI 0.64 to 1.01), both of which were secondary endpoints. Interestingly, the survival benefit of denosumab was observed only in patients who commenced denosumab concurrently with an aromatase inhibitor (for OS HR 0.41, 95% CI 0.32–0.73). However, the results of the trial were not formally significant, as they were reported after the negative analyses of DFS. Additionally, the 5-year adjuvant denosumab did not show any disease-related improvements across the subgroups of the large D-CARE trial (*n* > 4,500), where the primary endpoint was bone metastasis-free survival (HR 0.97, 95% CI 0.82–1.14) [81]. Osteonecrosis of the jaw occurred in 5% of the patients in the denosumab arm, compared to just 0.2% in the placebo group. Due to the formally negative findings from these studies, denosumab is not currently indicated as an adjuvant therapy, although ongoing research continues to investigate potential subgroups that may benefit from RANKL inhibition, such as *BRCA1* carriers [82].

### CDK4/6 inhibitors

Cyclin-dependent kinase 4/6 (CDK4/6) inhibitors abemaciclib, palbociclib, and ribociclib, which are approved for the treatment of metastatic HR+/HER2 breast cancer, have also been examined in the adjuvant setting (Table 3). While the phase III studies involving palbociclib did not show any improvement in invasive disease-free (iDFS) survival for stage II-III patients or those with residual disease after neoadjuvant chemotherapy, both abemaciclib and ribociclib have been approved for use in adjuvant treatment [71, 72, 83-86, 87].

**Table 3:** The trials evaluating CDK4/6 inhibitors in the adjuvant setting of HR+/HER2- breast cancer. iDFS was the primary endpoint in all studies mentioned in the table.

Trial acronym, NCT number	Main inclusion criteria	Number of randomized patients	Intervention arm	iDFS benefit	Median follow-up time	EMA and FDA approval
MonarchE, NCT03155997 [85]	Cohort 1: ≥ 4 ALN OR 1–3 ALN with grade 3 and/or tumor size ≥ 5 cm Cohort 2: 1–3 ALN and Ki-67 ≥ 20% (grade 1–2 and/or tumor size ≤ 5 cm)	5,637	Abemaciclib 150 mg twice daily for 2 years with SOC endocrine therapy	HR 0.67 (95% CI 0.59–0.76)	54 months	Approved (cohort 2 only by FDA)
NATALEE, NCT03701334 [86]	Stage II with high-risk features or stage III	5,101	Ribociclib 400 mg once daily days 1–21 of a 28-day cycle for 3 years with SOC endocrine therapy	HR 0.75 (95% CI 0.63-0.89)	33.3 months	Approved
PALLAS, NCT02513394 [84]	Stage II or III	5,796	Palbociclib 125 mg daily days 1–21 of a 28-day cycle for 2 years with SOC endocrine therapy	HR 0.96 (95% CI 0.81–1.14)	31 months	Not approved
PENELOPE-B, NCT01864746 [83]	Residual disease at surgery, with CPS-EG score ≥ 3 or 2 with ypN+	1,250	Palbociclib 125 mg daily days 1–21 of a 28-day cycle for 1 year with SOC endocrine therapy	HR 0.93 (95% CI 0.74–1.17)	42.8 months	Not approved

ALN: axillary lymph node; CI: confidence interval; CPS-EG: clinical pathological staging-estrogen receptor grading; HR: hazard ratio; iDFS: invasive disease-free survival; NCT: National Clinical Trial; SOC: standard of care; HER2: human epidermal growth factor receptor 2.

The most mature data comes from the MonarchE trial, which evaluated whether adding 2 years of abemaciclib to endocrine therapy could primarily improve iDFS [85]. The patients had a high risk of recurrence, with 65.3% having four or more metastatic axillary lymph nodes. Patients with one to three metastatic axillary lymph nodes were also included, but in these cases, tumors were either grade 3 differentiated or at least 5 cm in size. There was also Cohort 2 in the MonarchE trial, where the high-risk disease was defined by one to three metastatic axillary lymph nodes and a Ki-67 of at least 20%. However, these patients constituted only 9% of the total study population, and this proliferation-based risk stratification has not led to approval, for example, by the European Medicine Agency. In the latest update with 54 months of follow-up, iDFS improved by 7.9% points at 5 years (HR 0.67, 95% CI 0.59–0.76). Practically, at 5 years, for every 100 patients meeting the MonarchE criteria, 24 patients treated with only endocrine therapy experienced recurrence and abemaciclib prevented recurrence in 8 out of these 24 patients. The current number needed to treat figure remains around 13. Clinically importantly, the number of distant metastases was significantly reduced in the abemaciclib arm (HR 0.67, 95% CI 0.58–0.77), while follow-up for overall survival is still immature and statistically non-significant (HR 0.89, 95% CI 0.74–1.08).

As expected, neutropenia and diarrhea were the most common grade 3–5 adverse events in the MonarchE trial, occurring in 19.6 and 7.8% of the patients receiving abemaciclib, respectively. Notably, the overall quality of life was not compromised in the abemaciclib arm [87]. It is important to highlight that 6.5% of the patients completely discontinued both abemaciclib and endocrine therapy (ET), compared to only 1.1% in the ET-only arm, and these figures are likely much higher in real-world settings. Consistent with the findings in metastatic breast cancer, treatment efficacy was not compromised when the abemaciclib dose was reduced in response to side effects [90]. Interstitial lung disease was rare, with an incidence of 3.2% among abemaciclib-treated patients, but it should remain on clinicians’ minds. Pulmonary embolisms were reported in 28 patients (1.0%) in the abemaciclib arm and in 4 patients (0.1%) in the control arm. Therefore, it may be prudent to consider prescribing an aromatase inhibitor alongside OFS for premenopausal patients initiating abemaciclib, due to the pulmonary embolism risk associated with tamoxifen [39]. Real-world data on adjuvant abemaciclib is still limited; however, in a Japanese cohort of 374 patients, dose reductions occurred in 42% of the cases, which is comparable to 43.4% in MonarchE [88].

The follow-up from the ribociclib adjuvant trial, NATALEE, is shorter, but the final report of the iDFS results has recently been published, with a median follow-up of 33.3 months for the iDFS, the primary endpoint of the study [86]. At the data cut-off, all patients had completed ribociclib treatment. Ribociclib provided an iDFS benefit with an HR of 0.75 (95% CI 0.63–0.89) and also prolonged distant disease-free survival (DDFS) with a comparable effect. Similar to MonarchE, the difference between the study arms also in NATALEE seems to continue to show separation in the Kaplan-Meier curves after the discontinuation of the CDK4/6 inhibitor, suggesting a carry-over effect for the reduction of all recurrences and distant metastases. In comparison to MonarchE, patients in NATALEE received the CDK4/6 inhibitor for 3 years at a lower dose (400 mg once daily) than in the metastatic setting. Most notably, NATALEE included a much broader population of early breast cancer patients, including node-negative patients. The side effects in NATALEE aligned with the previously established safety profile of ribociclib. The rate of early discontinuation of the CDK4/6 inhibitor due to adverse events was comparable in both trials, 20% in NATALEE and 18% in MonarchE [85, 86]; however, in clinical practice, these figures are likely to be substantially higher. As both tamoxifen and ribociclib may cause the prolongation of QT interval, it may be advisable to consider an aromatase inhibitor alongside OFS for premenopausal patients initiating ribociclib.

The definite reasons for negative palbociclib adjuvant studies remain unclear. One explanation is the less optimal pharmacodynamic properties of palbociclib, as palbociclib phase III studies have failed to show overall survival benefit also in the metastatic setting, in contrast to several phase III studies with abemaciclib and ribociclib [90–95]. Again, the largest palbociclib adjuvant trial, PALLAS, enrolled also patients with lower-risk disease (e.g. node-negative tumors without additional high-risk features), potentially diluting any benefit, while PENELOPE-B was a smaller post-neoadjuvant trial with only 1 year of palbociclib treatment (vs. 2 years in MonarchE and 3 years in NATALEE), which could have been insufficient to impact long-term outcomes [86, 99]. Furthermore, although PALLAS had high rates of dose reductions and early discontinuations (45% of patients stopped palbociclib by 2 years due to toxicity), a landmark analysis showed no difference in iDFS even among patients who received most or all of the planned doses, indicating that inadequate drug exposure alone was not responsible for the lack of efficacy [84].

The introduction of abemaciclib and ribociclib in the adjuvant setting presents several clinically significant perspectives. In large Western retrospective studies, it is estimated that 9.5 to 18% of early breast cancer patients may be eligible for adjuvant treatment with abemaciclib, while 33 to 43% would meet the NATALEE criteria for ribociclib adjuvant therapy [96–98]. Given their toxicity, current lack of overall survival benefit, the need for frequent blood test (and ECG) monitoring, and associated costs, there is an urgent demand for predictive factors for both abemaciclib and ribociclib. Current strategies for selecting the high-risk population for CDK4/6 inhibitor adjuvant therapy rely solely on anatomical stage, grade, and proliferation, often leading to overtreatment, while a significant proportion of the patients still experiences relapse despite the intensified therapy. To address this issue, several ongoing studies are randomizing patients to either endocrine therapy alone or in combination with CDK4/6 inhibitor treatment, based on postoperative ctDNA, disseminated breast cancer cells of bone marrow, or changes in Ki-67 during induction therapy (NCT03285412, NCT04567420, NCT04985266, NCT04841148). In a small subanalysis from MonarchE, all patients (*n* = 42) who were ctDNA-positive at 24 months, experienced recurrence during the follow-up, while only 21.1% of the persistently ctDNA-negative patients (*n* = 133) had an iDFS event during the follow-up [99]. In other words, patients with no ctDNA seem to carry somewhat average prognosis, while ctDNA-positivity at 2 years indicates a dismal prognosis. Unfortunately, there are no known means to decrease the recurrence risk of the latter patients. Currently, for example, neither RNA sequencing nor molecular profiling has revealed any predictive factors from the MonarchE population, whereas such analyses have not yet been published from the NATALEE population. The effectiveness of reinitiating CDK4/6 inhibitors in the metastatic setting after prior adjuvant therapy, at least in the patients with a short disease-free interval, remains so far also unclear, and can be questioned. However, novel CDK4-specific inhibitors are currently studied in the late-phase in clinical trials, with the aim to overcome conventional resistance mechanisms to the currently used CDK4/6 inhibitors (e.g. NCT06105632, NCT06760637).

Finally, recent changes in the surgical treatment of the axilla may pose challenges when interpreting results from several adjuvant trials, including MonarchE and NATALEE. Axillary nodal involvement in at least four lymph nodes was a major inclusion criterion in MonarchE, and N2–N3 patients were also included in NATALEE. In clinical practice, identifying four metastatic axillary lymph nodes necessitates axillary evacuation. Following the publications of ACOSOG Z0011 and AMAROS, the number of axillary evacuations has considerably decreased [100, 101]. According to the *post hoc* analysis of the phase III SENOMAC trial, axillary evacuations would need to be performed in 104 patients to prevent one invasive recurrence if patients were treated with abemaciclib and endocrine therapy [102]. Based on calculations from this large dataset, these 104 axillary evacuations would lead to severe or very severe impairments in arm function for nine patients. In line with this, two European retrospective cohort studies indicated that after a positive sentinel node biopsy, at least four metastatic axillary lymph nodes are found in 12 to 13% of HR+/HER2- patients. This suggests that 1,000 patients would need to undergo axillary evacuation to diagnose 120 patients with N2-level lymph node involvement and subsequently to prevent four invasive recurrences with abemaciclib adjuvant treatment [103, 104]. In summary, increasing axillary evacuations cannot be recommended for diagnosing more patients with N2–N3 involvement and, therefore, for identifying candidates for abemaciclib or ribociclib adjuvant treatment.

### PARP inhibitors

About two-thirds of breast cancers associated with pathogenic germline variants in *BRCA1* show triple-negative subtype, while a HR+/HER2- subtype is identified in 60–70% of patients with pathogenic germline variants of the *BRCA2* gene [105]. In the phase III OlympiA trial, women with germline *BRCA1/2* mutations were randomized after curative-intent surgery and at least six cycles of (neo)adjuvant treatment that included anthracyclines and/or taxanes to receive either a placebo or the poly ADP ribose polymerase (PARP) inhibitor olaparib for 1 year [22, 139]. Among the patients, 17.7% (*n* = 325) had the HR+/HER2- subtype. The studied population was notably high risk, with the primary inclusion criteria for the HR+/HER2- subgroup being either at least four metastatic lymph nodes after upfront surgery or no pathological complete remission after neoadjuvant therapy, along with a clinical pathological staging-estrogen receptor grading (CPS + EG) score of at least 3 [106]. The primary endpoint, iDFS, was 82.7% in the olaparib arm and 75.4% in the placebo arm at 4 years (absolute difference 7.3%, 95% CI 3.0–11.5%). Clinically importantly, both DDFS and overall survival (4-year event-free rate of 89.8% vs. 86.4%) indicated significant improvement with olaparib [22, 139]. There was no difference in patient outcomes between the HR+/HER2- subtype and the overall study population. In a subsequent -quality-of-life analysis, olaparib did not significantly increase fatigue, but nausea and vomiting were notably more pronounced in the olaparib-treated patients [107]. Recent guideline changes have expanded the recommendations for germline *BRCA1/2* testing to include women aged ≤ 65 years if they have a history of breast cancer [108]. These recommendations are not solely based on the overall survival advantage of olaparib, but they also aim to more accurately identify *BRCA1/2* carriers, which could improve the prognosis of carriers through risk-reducing surgeries and more -tailored surveillance with magnetic resonance imaging [109–111].

Also *BRCA1/2* carriers with HR+/HER2- breast cancer seem to benefit from abemaciclib treatment similar to the non-carriers [112]. Concurrent treatment with abemaciclib and olaparib cannot be recommended, as their side effect profiles overlap, including frequent bone marrow suppression. In the MonarchE trial, recruitment was permitted up to 16 months after surgery; therefore, sequential treatment involving 1 year of olaparib followed by 2 years of abemaciclib could be considered for the selected patients with high-risk HR+/HER2- early-stage breast cancer and germline *BRCA1/2* mutation. However, there is no prospective evaluation on this, as *BRCA1/2* mutation carriers were excluded from MonarchE and no patients receiving adjuvant abemaciclib therapy participated in the OlympiA trial. While acknowledging the caveats of cross-trial comparisons, it can be stated that olaparib (and also bisphosphonates, with caution) is currently the only medication demonstrating an overall survival benefit after curative surgery and (neo)adjuvant therapy in HR+/HER2- breast cancer. Although pathogenic germline *PALB2* variant carriers have also responded well to PARP inhibitors in metastatic breast cancer, the low incidence of *PALB2* mutations will make it challenging to demonstrate the benefit in the adjuvant setting [113].

## Emerging treatment targets

Beyond novel endocrine therapies, PARP inhibitors, and CDK4/6 inhibitors, new drug classes targeting HR+/HER2- breast cancer in the adjuvant setting are scarce. Currently, perioperative immuno-oncological treatment is not approved for HR+/HER2- breast cancer. However, this may change soon based on the results of the phase III KEYNOTE-756 trial, which has reported an 8.5% point improvement when pembrolizumab was added to chemotherapy in the perioperative treatment of high-risk HR+/HER2- breast cancer [114]. Early evidence from prospective studies also shows the potential feasibility of combining a programmed death ligand-1 (PD-L1) inhibitor with olaparib [115, 116]. A phase II study combining another PD-L1 inhibitor, nivolumab, with abemaciclib in HR+/HER2- breast cancer patients resulted in severe and often prolonged immune-related hepatitis in most patients [117]. Antibody conjugates, such as trastuzumab deruxtecan and sacituzumab govitecan, have shown their efficacy in metastatic breast cancer and are now being tested in the neoadjuvant setting [74, 118–121]. However, no studies focusing solely on the pure adjuvant setting are currently underway.

## Discussion

Selecting the optimal post-chemotherapy adjuvant treatment for patients with HR+/HER2- breast cancer requires balancing the highest level of evidence-based care with individual patient-specific factors and system-level constraints. The first consideration for a clinician is disease risk: while all high-risk patients should receive endocrine therapy, usually for 7–10 years, the options enhancing it include CDK4/6 inhibitors, bisphosphonates, and olaparib. In real-world decision-making, toxicities and patient preferences must be weighed alongside efficacy. For example, ovarian suppression induces menopausal symptoms that can substantially decrease the quality of life, leading to treatment discontinuation in more than one-third of the patients [74]. In phase III adjuvant trials with CDK4/6 inhibitors, 20–30% of the patients discontinued treatment due to toxicity or other issues, and the real-world adherence is likely much poorer [85, 86].

On a system level, the utilization of post-chemotherapy adjuvant treatment is strongly influenced by variations in healthcare infrastructure, drug availability, and socioeconomic considerations [122]. Even in the high-income countries, reimbursement policies for breast cancer drugs vary a lot [123]. Core endocrine treatments, tamoxifen, and more recently also aromatase inhibitors, are off-patent and widely available globally, but subtle disparities persist [124]. Although genomic assays are often unavailable in low-income countries, clinical tools such as CTS5 may also inform higher risk without additional cost [48–50]. Inexpensive tamoxifen is the mainstay in the low-income countries, sometimes even for those who might benefit from aromatase inhibitors and OFS. The cost and logistics of the monthly injections of OFS can be challenging to implement in lower-income countries, and as a result, some oncologists in resource-limited environments resort to surgical oophorectomy, despite its irreversible side effects [125]. The disparities in access to bone mineral density monitoring and supportive care when on aromatase inhibitor therapy should not be underestimated. The adherence to endocrine treatment is also clearly suboptimal in patients living in developing countries, which is associated with increased risk of death [126–128]. For access to high-cost medications, such as olaparib and CDK4/6 inhibitors, the situation is much worse in low- and middle-income countries. Three years of ribociclib cost around $500,000 in the United States, with a $27 million expenditure to avoid one iDFS event [129]. Without price reductions or special access programs, these drugs are largely out of reach from most of the breast cancer patients globally. The patients using these drugs also require frequent hematological monitoring, which, along with an overburden healthcare is difficult to sustain in resource-limited systems. Some clinical guidelines, such as St. Gallen and ASCO, have made specific resource-sparing recommendations, and several countries created tiered guidelines (basic, limited, enhanced, maximal resources) aligning with the WHO’s Global Breast Cancer Initiative [130–134].

## Conclusions

The prognosis for HR+/HER2- breast cancer has significantly improved over the past few decades and partly due to the systematic screening programs, the proportion of high-risk patients is decreasing [135, 136]. At the same time, several novel adjuvant treatments have been introduced, and most patients are now eligible for non-endocrine treatments after chemotherapy. However, there are considerable adherence issues even with the 5-year endocrine treatment, primarily due to toxicity and access to these treatments is globally inequal [59–61, 126]. Therefore, there is a strong need to more accurately identify patients who would benefit most from the adjuvant treatments, as well as those who could be spared from their toxicity. Strategies such as ctDNA monitoring may enable treatment de-escalation in some high-risk patients. Discovering new predictive factors may depend much on academic research, including the initiation of studies with non-inferiority design. In the near future, with the introduction of perioperative immunological treatments, novel CDK4-specific inhibitors, and more targeted endocrine therapies, a significant proportion of previously high-risk HR+/HER2- breast cancers will likely show a relatively favorable prognosis.

## Data Availability

Not applicable.
